# Hydraulic Traits Constrain Salinity-Dependent Niche Segregation in Mangroves

**DOI:** 10.3390/plants14121850

**Published:** 2025-06-16

**Authors:** Haijing Cheng, Yinjie Chen, Yunhui Peng, Mi Wei, Junfeng Niu

**Affiliations:** 1 College of Life Sciences and Oceanography, Shenzhen University, Shenzhen 518060, China; 2 The Wetland Park of Overseas Chinese Town, Shenzhen 518052, China; 3 School of Agriculture, Shenzhen Campus, Sun Yat-Sen University, Shenzhen 518107, China

**Keywords:** mangroves, embolism, isohydry and anisohydry, hydraulic safety margin, specific leaf area, wood density

## Abstract

To understand the mechanisms underlying species assemblage along salt gradients in intertidal zones, we measured the xylem hydraulic vulnerability curves (*HVC*s), leaf water potential (*ψ*), stomatal conductance (*g*_s_), specific leaf area (*SLA*), and wood density (*WD*) for six mangrove species of *Avicennia marina*, *Bruguiera gymnorrhiza*, *Aegiceras corniculatum*, *Kandelia obovata*, *Sonneratia apetala*, and *Sonneratia caseolaris*. We found the following: (1) *A. marina* and *B. gymnorhiza* had the most negative *P*_50_ (water potential at which 50% of hydraulic conductivity was lost), while *S. caseolaris* and *S. apetala* had the least negative *P*_50_, indicating different resistance to embolism in xylem; (2) *P*_50_ and *P*_88_ (water potential at which 88% of hydraulic conductivity was lost) declined with increasing salinity from the onshore to offshore species, as their water regulation strategy meanwhile transitioned from isohydry to anisohydry; (3) *B. gymnorhiza* had smaller *SLA* but larger hydraulic safety margin (*HSM*), implying potentially higher capacity of water retention in leaves and lower risk of hydraulic failure in xylem. These results suggest that hydraulic traits play an important role in shaping the salt-driven niche segregation of mangroves along intertidal zones. Our research contributes to a more comprehensive understanding of the hydraulic physiology of mangroves in salt adaption and may facilitate a general modeling framework for examining and predicting mangrove resilience to a changing climate.

## 1. Introduction

Mangroves grow in tidal, saline wetlands along tropical and subtropical coasts worldwide [1]. They provide important habitats for terrestrial and marine animals including birds, insects, mammals, reptiles, and shellfish, and have the function of improving water quality and protecting dikes from waves and storms [2]. Salinity is one of the most important factors shaping the morphology and typology of mangrove ecosystems, largely accounting for the niche differentiation among sympatric species in coastal regions [3,4]. Therefore, it is necessary to perform interspecies comparative studies to better understand the competitive advantages of different mangroves colonizing along a varying salt gradient.

High and fluctuating salinity may cause water deficit for mangrove trees [5]. To extract water efficiently from saline substrates, mangroves have to maintain a lower internal osmotic potential [6]. Meanwhile, many mangrove species have evolved physiological adaptations similar to those of xerophytes for water conservation [1,7]. Global warming and intensifying heat waves may boost evapotranspiration and exacerbate hydraulic tensions, leading to embolism in mangrove xylem vessels. That could in turn induce stomatal closure, limit carbon assimilation, and affect plant survival and growth [8]. Thus, it is important to improve our understanding of mangrove xylem hydraulics so that we can better predict and assess their responses to future climate scenarios.

Water potential, inducing a specific percentage decrease in hydraulic conductivity (*P*_x_), is often used to characterize xylem embolism resistance [9]. Salt tolerance in mangroves can also be described in the same way if its ionic effect was neglected and only the osmotic effect was considered [7]. *P*_12_, *P*_50_, and *P*_88_, representing xylem water potentials corresponding to the loss of hydraulic conductivity by 12%, 50%, and 88%, respectively, are the most frequently adopted indexes. *P*_50_ was considered as a key indicator for plant tolerance to stress and extensively used in hydraulic safety or vulnerability assessments [10,11]. *P*_12_, *P*_50_, and *P*_88_ are all generally extracted from hydraulic vulnerability curves (*HVC*s). However, *HVC*s also bear information about the sensitivity of conductivity loss (*S*_x_) to water potential changes, and this *S*_x_ has so far been largely underappreciated.

The hydraulic safety margin (*HSM*) is another important indicator for plant hydraulic risk, and is commonly quantified as the difference between the minimum water potential (*ψ*_min_) experienced by plants under natural conditions and their *P*_50_ [12,13]. A large positive *HSM* means a relatively low risk of embolism, and thus a low level of hydraulic failure; by contrast, a small positive or even negative *HSM* may imply potential hydraulic threat [10,14]. It can be speculated that mangroves are different in *HSM*s due to their different *P*_50_ and *ψ*_min_.

Plant functional traits can track environmental changes, affect plant fitness, and play an important role in shaping species co-occurrence patterns within ecosystems [15,16]. Both specific leaf area (*SLA*) and wood density (*WD*) are widely used functional traits. *SLA* reflects plant adaptive response to optimize light capture in leaves [17]; meanwhile, *WD* accounts for a tradeoff between carbon storage and hydraulic efficiency in xylem [18,19]. Mangrove plants can adjust leaf size and thickness to improve their adaptability to salt [20]. It has also been reported that the mangrove species of *Avicennia germinan*, which inhabits the most seaward saline environment, had the highest *WD* on the Amazon coast [21]. However, there is still a lack of research that can directly link *SLA* and *WD* to mangrove niche partitioning along salinity gradients in intertidal zones.

Plant tolerance to drought and salt highly depends on leaf stomatal behaviors: isohydric species close stomata rapidly, while anisohydric species tend to keep stomata open and maintain water transpiration even under more negative osmotic potentials [22,23]. More generally, plants coexist along a continuous spectrum from isohydry to anisohydry, with the former being resistant to embolism and the latter being tolerant of embolism [24]. If salinity increases (and thus water potential decreases) from the onshore to offshore habitats, the stomatal water regulation strategies of mangroves should also transition from isohydry to anisohydry along this coastal region.

In the present study, we selected six representative mangrove species, including *Sonneratia caseolaris*, *Sonneratia apetala*, *Kandelia obovate*, *Bruguiera gymnorrhiza*, *Aegiceras corniculatum*, and *Avicennia marina* in Shenzhen Overseas Chinese Town National Wetland Park. We measured leaf water potential (*ψ*) dynamics, stomatal conductance (*g*_s_), *WD*, and *SLA* and we constructed xylem *HVC*s, analyzed *P*_x_ and *S*_x_, and calculated the *HSM* for each species. We hypothesized the following: (1) salt resistance varied among mangroves distributed along salinity gradient from the onshore to offshore sites; (2) water regulation strategies changed from isohydry to anisohydry in mangroves as substrate salinity increased toward the offshore habitats; (3) *HSM* and *WD* tend to be low and *SLA* tend to be high for the onshore species of *S. caseolaris* and *S. apetala*, while the offshore species of *B. gymnorrhiza* and *A. marina* should have high *HSM* and *WD*, but low *SLA*. We aim to elucidate the underlying mechanisms that constrain mangrove distribution along salt gradients in intertidal zones. The results are meaningful for the monitoring, protection, reestablishment, and restoration of coastal mangrove ecosystems.

## 2. Materials and Methods

### 2.1. Field Site and Study Species

This study was conducted in the Overseas Chinese Town (OCT) Wetland Park (113.99 N, 22.53 E) in Shenzhen. It is the only coastal mangrove wetland in the urban hinterland in China. It covers an area of about 68.5 ha, and has a subtropical monsoon climate with mean annual precipitation and air temperature of 1823 mm and of 24 °C, respectively. The regional dominant mangrove species include *Sonneratia caseolaris*, *Sonneratia apetala*, *Kandelia obovate*, *Bruguiera gymnorrhiza*, *Aegiceras corniculatum* and *Avicennia marina*, distributing sequentially along the intertidal zone.

*S. caseolaris* and *S. apetala* occupy the most inland estuary area (flooded only during high tide) with continuous freshwater replenishment from an inner lake, leading to a relatively low substrate salinity of between 3.5 and 6.2‰. *A. marina* is located exclusively in the most seaward site and is immersed throughout the year in seawater with a changing salinity from 17.3‰ to 24.5‰. The distribution of *A. corniculatum* is partially overlapped with that of *K. obovate* and *B. gymnorrhiza* (with a substrate salinity of 9.1–15.4‰). They are geographically closer to *A. marina* than *S. caseolaris* and *S. apetala*, and their roots are also immersed in seawater most of the time. All salinity was measured monthly with a YSI Pro30 field conductivity meter (YSI Inc., Yellow Springs, OH, USA). For each species, we selected four to six individual trees of similar physical status to carry out measurements. All measurements were taken in trees that represent typical site characteristics.

### 2.2. Leaf Water Potential and Stomatal Conductance

*ψ* (leaf water potential) was measured using a Model 1505D-EXP pressure chamber (PMS Instrument Company, Corvallis, OR, USA), and *g*_s_ (stomatal conductance) was screened by a SC-1 leaf porometer (METER, San Francisco, CA, USA). To compensate for the possible effect of the sensor head on *g*_s_, the porometer took readings within 30 s [25]. For the measurement of *ψ*, leaves were carefully excised, sealed in plastic bags, and stored in a portable icebox for 20 min. The equilibrated leaves were taken out and inserted into the chamber lid with the petiole cut surface protruding out through a gasket. The lid is replaced, holding the leaf blade within the sealed chamber, and the chamber is pressurized slowly (0.01 Mpa s^−1^) until the petiole cut surface is wetted by the rising sap meniscus. The ‘balancing pressure’ applied has exactly compensated for the negative pressure in the xylem water column [26]. Four to six fully expanded, healthy, and sun-exposed leaves for each tree and four to six individual trees for each species were sampled. Measurements were taken every two hours in the field from 5:00 to 19:00 on clear days over the summer growing season in 2020.

### 2.3. Hydraulic Vulnerability Curves

Sun-exposed terminal branches (about 1–1.2 m in length) were harvested from the upper canopy positions of the sample trees at predawn (5:00–6:00 am), and transported immediately to the laboratory in black plastic bags to prevent moisture loss [27]. In the laboratory, a stem section (about 3–5 cm in length) was cut off from the bottom of each branch sample under water to avoid the inclusion of embolized vessels [28]. Stem segments measuring 6–8 cm in length and 0.8–1 cm in diameter were then prepared, and both the basal and distal ends were trimmed using a razor blade. The bark of the central part of each stem segment was removed and the sapwood was slightly notched using sharp blade to facilitate air seeding [29].

Embolism was induced by injecting air into a steel sleeve (4 cm in length) at different pressure levels. The sleeve was connected to a Model 1505D-EXP pressure chamber, allowing the injection of compressed nitrogen gas. Stem segment samples were inserted into the sleeve with two ends protruding out. Air was injected for 5 min at each of the following pressure levels: 1.0, 2.0, 3.0, 4.0, 5.0, 6.0, 7.0, 8.0, 9.0, and 10.0 Mpa. The injected segments were allowed at least 30 min to equilibrate after each pressurization until no more bubbles came out of the xylem [30].

Post-injection hydraulic conductivity (*K*) was assessed with a commercial XYL’EM apparatus (Bronkhorst, Montigny-les-Cormeilles, France). At least 5 subsamples (0.8–1 cm in diameter) measuring 2 cm in length were excised under water, and their proximal ends were connected to the tubing system of XYL’EM. The flow was measured by a high-resolution liquid mass flow meter, and *K* (kg m^−1^ s^−1^ Mpa^−1^) was calculated as the ratio of flow rate to pressure gradient (6 kPa). Initial hydraulic conductivity (*K*_ini_) was measured using the unpressured stem samples. They were then flushed with ultrapure water at 0.15 Mpa for 30 s and remeasured to determine the maximal conductivity (*K*_max_). The percentage loss of hydraulic conductivity (*PLC*) at each pressure level was calculated as (Equation (1)):

(1) $$PLC=\frac{\left(K_{\max}-K\right)}{K_{\max}}\times 100\%$$

*HVCs* (hydraulic vulnerability curves) were constructed by fitting *PLC* with xylem water potentials (equal in number to the injection pressure but with opposite signs) using an exponential–sigmoidal function (Equation (2)). *P*_12_, *P*_50_, and *P*_88_ (water potential corresponding to 12%, 50% and 88% loss in hydraulic conductivity) were assessed from the *HVC* for each tree species. *P*_12_ measures the initiation of embolism and *P*_88_ signifies complete embolism. *P*_50_ is the most commonly used index of embolism resistance, at which *HVCs* are steepest [31]. We also assessed the slope (S_x_) of *HVCs* at *P*_12_, *P*_50_ and *P*_88_ (*S*_12_, *S*_50_ and *S*_88_). (Table 1) These parameters represent the sensitivity of conductivity loss to water potential changes in the xylem vessels. The 95% confidence intervals (CIs) for *P*_x_ and *S*_x_ were estimated by the bootstrap resampling approach with replacement (N = 1000) [32].

(2) $$PLC=\frac{100}{1+\exp(a(\psi -b))}$$

where *a* is the slope at the inflection point and *b* is the *ψ* value of the inflection point of the fitting curve. It can be easily proved that *b* equals *P*_50_ [33]. The *HSM* (hydraulic safety margin) was computed as the difference between the minimum leaf water potential (*ψ*_min_) and *P_50_* [34].

### 2.4. Specific Leaf Area and Wood Density

Sun-exposed healthy leaves were taken from the same sample trees for *g*_s_ and *ψ* measurements. Fresh leaves were scanned and weighted in field as soon as they were collected, then put in labelled envelopes and brought to the laboratory. Leaf areas were analyzed using ImageJ (Version 1.54). Leaves were then oven dried at 75 °C for 72 h, and dry mass was determined by an analytical balance with an accuracy of 0.0001 g (Thermo Fisher Scientific Inc., Waltham, MA, USA). *SLA* (specific leaf area) was calculated as the ratio of leaf area to dry mass [35].

Stem segments about 1.5 cm in length and 0.8–1.0 cm in diameter were cut from the harvested stem branches. Barks were removed, and fresh volume (*FV*) was determined with the water-displacement method (available online: https://prometheusprotocols.net). Stem segments were then oven-dried at 80 °C until constant dry weight (*DW*) was obtained (Thermo Fisher Scientific Inc., Waltham, MA, USA). *WD* (wood density) was calculated as the ratio of *DW* to *FV*.

### 2.5. Statistical Analyses

The *HVC* was fitted using the fitplc package [32] in R-4.2.1. Data were analyzed with SPSS 20 and visualized in origin 2023. Normality and homogeneity of variance were examined before analyses. One-way analyses of variance (ANOVA) with Duncan’s multiple-range tests were performed to test trait differences among tree species. Relationships between two quantitative variables were assessed based on Pearson correlation tests and linear regression models. Statistical analyses were considered significant if *p* < 0.05.

## 3. Results

### 3.1. Hydraulic Vulnerability and Sensitivity

**Figure 1:**
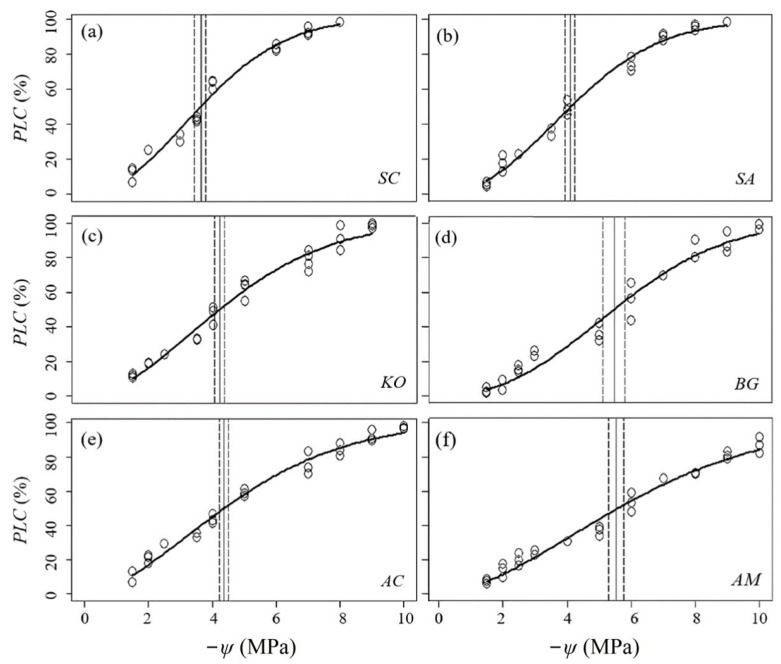
Percentage loss of xylem hydraulic conductivity (*PLC*, %) as a function of xylem water potential (*ψ*, −Mpa) for the six mangrove species. The vertical solid lines indicate the *ψ* corresponding to 50% loss of hydraulic conductivity (*P*_50_) and vertical dashed lines indicate the bootstrap (N = 1000) estimated 95% confidence intervals for *P*_50_. (**a**) *Sonneratia caseolaris*; (**b**) *Sonneratia apetala*; (**c**) *Kandelia obovata*; (**d**) *Bruguiera gymnorrhiza*; (**e**) *Aegiceras corniculatum*; (**f**) *Avicennia marina*.

*PLC* (percentage loss of hydraulic conductivity) fitted well with the chamber pressures adopted to induce cavitation in branch xylem (Figure 1). Decreased *ψ* (leaf water potential) led to increased embolism. *P*_x_ (water potential corresponding to x% loss in hydraulic conductivity) varied significantly (*p* < 0.05) among the six mangroves, and was generally lower (more negative) in the offshore species of *Bruguiera gymnorrhiza* and *Avicennia marina*. *A. marina* also had a significantly lower (more negative) *P*_88_ (water potential corresponding to 88% loss in hydraulic conductivity) than *B. gymnorrhiza*, while the differences of *P*_12_ (water potential corresponding to 12% loss in hydraulic conductivity) and *P*_50_ (water potential corresponding to 50 % loss in hydraulic conductivity) were not significant between them. For the onshore species of *Sonneratia caseolaris* and *Sonneratia apetala*, *P*_50_ was significantly higher (less negative) in the former than in the latter, while the differences of *P*_12_ and *P*_88_ were not significant. The mangrove species of *Kandelia obovata* and *Aegiceras corniculatum* tended to have intermediate *P*_x_ (Table 2).

*S*_x_ (slope of hydraulic vulnerability curves) tend to increase first, reach their maximum at *S*_50_ (the slope of the hydraulic vulnerability curve at *P*_50_), and then decrease as embolism percentages (*x*) increase. *S*_x_ varied significantly (*p* < 0.05) among the six mangroves, and was generally larger in the onshore species of *S*. *caseolaris* and *S*. *apetala*. *S*. *caseolaris* had a significantly larger *S*_12_ (the slope of the hydraulic vulnerability curve at *P*_12_) than *S*. *apetala*, while the differences of *S*_50_ and *S*_88_ (the slope of the hydraulic vulnerability curve at *P*_88_) were not significant between them. For the offshore species of *B. gymnorrhiza* and *A. marina*, *S*_50_ and *S*_88_ were significantly larger in the former than in the latter, while the difference of *S*_12_ was not significant. The mangrove species of *K. obovate* and *A.* *corniculatum* tended to have intermediate *S*_x_ (Table 2).

### 3.2. Stomatal Conductance and Water Potential

Daily changes in *g*_s_ (leaf stomatal conductance) showed a unimodal pattern for the onshore species of *S. caseolaris* and *S.apetala*, with the maximum *g*_s_ occurring at about 10:00 am and then decreasing all the way to below 130 mmol m^−2^ s^−1^ at 17:00. By contrast, *K. obovate*, *B. gymnorrhiza*, *A. corniculatum*, and *A. marina* had bimodal *g*_s_ dynamics, also peaking at about 10:00 am, but decreasing at noon and then increasing after 14:00 for a period of time, consistently maintaining above 200 mmol m^−2^ s^−1^ throughout the whole day of the measurement (Table 3).

While *g*_s_ was generally larger in *A. corniculatum* and *A. marina* than in *S. caseolaris*, *S. apetala*, *K. obovate*, and *B. gymnorrhiza*, its variation was significantly larger in *S. caseolaris* and *S. apetala* than in the other four species. During the measurement period from 6:00 to 17:00, the variation of *g*_s_ was largest in *S. caseolaris* with a coefficient of variation (*CV* = standard deviation/mean) of 27% and a changing range (△*g*_s_, the difference between the maximum and minimum *g*_s_) of 178.5 mmol m^−2^ s^−1^. By contrast, *g*_s_ varied least in *B. gymnorrhiza* with a *CV* of 10% and △*g*_s_ of 99.5 mmol m^−2^ s^−1^. For other species, the *CV* (14–23%) and △*g*_s_ (108–124 mmol m^−2^ s^−1^) fell between *S. caseolaris* and *B. gymnorrhiza* (Table 3).

**Figure 2:**
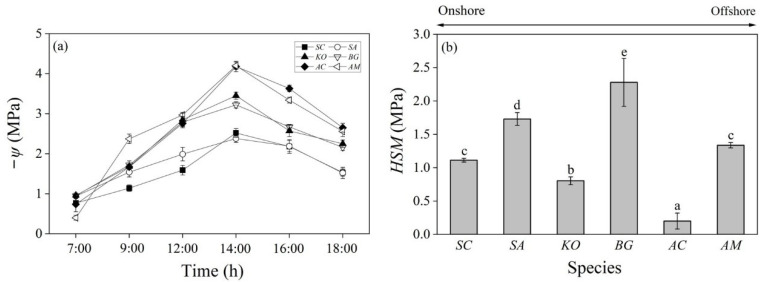
Diurnal leaf water potential (*ψ*, −Mpa) (**a**) and hydraulic safety margins (*HSM*s, Mpa) (**b**) of the six mangrove species. The *HSM* was calculated as the difference between the minimal leaf water potential (*ψ*_min_) under field conditions and the water potential corresponding to 50% loss of hydraulic conductivity (*P*_50_). Different letters indicate significant differences among species (mean ± SD, *n* = 3). *SC*: *Sonneratia caseolaris*; *SA*: *Sonneratia apetala*; *KO*: *Kandelia obovata*; *BG*: *Bruguiera gymnorrhiza*; *AC*: *Aegiceras corniculatum*; *AM*: *Avicennia marina*.

*ψ* (leaf water potential) decreased from 7:00 to 14:00 and then increased gradually until 18:00. *ψ*_pre_ (the predawn *ψ*) was below 0 Mpa, but above −1 Mpa for all six mangrove species. *A. corniculatum*, *A. marina*, and *S. caseolaris* had significantly higher (less negative) *ψ*_pre_ than *S. apetala*, *K. obovate*, and *B. gymnorrhiza*, with *A. marina* having the highest (least negative) *ψ*_pre_ of −0.40 Mpa. Meanwhile, *A. marina* and *A. corniculatum* also had the lowest (most negative) *ψ*_min_ (the minimum *ψ*), significantly lower (more negative) than that of the other four species. *ψ* was consistently lower (more negative) in *A. marina* and *A. corniculatum* than in other species from 14:00 to 18:00. By contrast, *S. caseolaris* and *S. apetala* had significantly higher (less negative) *ψ* throughout the whole day except for the predawn time of between 06:00–07:00 (Figure 2a and Table A1). Due to the similar *ψ*_pre_, but contrasting *ψ*_min_, the range of *ψ* (△*ψ*) was significantly wider in *A. marina* (3.80 Mpa) and *A. corniculatum* (3.44 Mpa) than in other species (1.44–2.49 Mpa) (Table A1).

### 3.3. Hydraulic Safety Margin

The mangrove species of our present study all had a positive *HSM* (hydraulic safety margin), meaning that their *ψ*_min_ was higher (less negative) than the *P*_50_ calculated from *HVC*s. The *HSM* was significantly wider in *B. gymnorrhiza* than in other mangroves. *S. apetala* also had a significantly wider *HSM* than *S. caseolaris*, *K. obovate*, *A. corniculatum*, and *A. marina*. *S. caseolaris* and *A. marina* had similar *HSM*s of between 1 and 1.5 Mpa, while *A. corniculatum* had the narrowest *HSM* (0.2 Mpa). The *HSM* of *K. obovate* was significantly wider than that of *A. corniculatum*, but significantly narrower than those of *S. apetala*, *B. gymnorrhiza*, and *A. marina* (Figure 2b).

### 3.4. Specific Leaf Area and Wood Density

**Figure 3:**
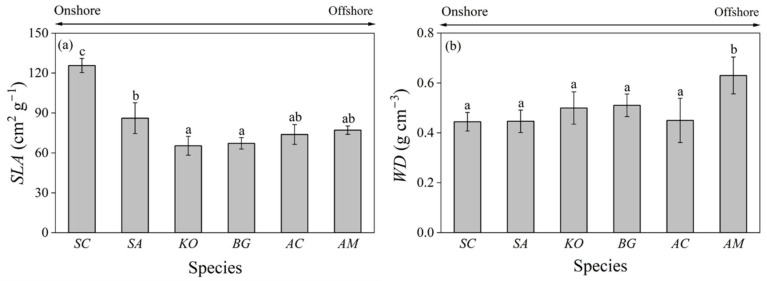
Specific leaf area (*SLA*, cm^2^ g^−1^) (**a**) and wood density (*WD*, g cm^−3^) (**b**) of the six mangrove species. Different letters indicate significant differences among species (mean ± SD, *n *= 3–15). *SC*: *Sonneratia caseolaris*; *SA*: *Sonneratia apetala*; *KO*: *Kandelia obovata*; *BG*: *Bruguiera gymnorrhiza*; *AC*: *Aegiceras corniculatum*; *AM*: *Avicennia marina*.

*S. caseolaris* and *S. apetala* had a significantly larger *SLA* (specific leaf area) than *K. obovate* and *B. gymnorhiza*. The *SLA* of *S. caseolaris* was nearly twice that of *K. obovate* (125.68 vs. 65.36 cm^2^ g^−1^). The *SLA* of *A. corniculatum* and *A. marina* also tended to be larger than those of *K. obovate* and *B. gymnorhiza*, but was smaller in comparison to those of *S. caseolaris* and *S. apetala*; yet, none of these differences were statistically significant (Figure 3a).

*WD* (wood density) was significantly larger in *A. marina* (0.63 g cm^−3^) than in other five mangrove species. Although *K. obovate* and *B. gymnorhiza* also tended to have larger *WD* than *S. caseolaris*, *S. apetala*, and *A. corniculatum*, the differences among them were not statistically significant (Figure 3b).

## 4. Discussion

Mangroves are periodically inundated by tides, and the high salinity of seawater may pose a great challenge to their growth [36]. Although the ion toxicity of salt is commonly ion-specific, its osmotic stress on plants is intrinsically similar to that of drought. Mangroves may therefore experience a physiological drought, and be potentially at the risk of drought-induced cavitation and embolism [37]. If that were the case, there would be trait differentiation among mangrove species distributed along salinity gradients. Traits relate to plant form and function, and the functional traits in turn affect plant responses to climate change and abiotic stresses [11,38]. The covariation between plant functional traits and various environmental factors has been well established in the literature [39,40,41].

*SLA* (specific leaf area) and *WD* (wood density) have been related to growth performance of trees and forests in response to drought across biomes. Species in arid environments tend to have low *SLA* and high *WD* [42,43]. In the present study, we found that *SLA* was significantly larger (*p* < 0.05) in the onshore species of *Sonneratia caseolaris* and *Sonneratia apetala*, while *WD* was significantly larger (*p* < 0.05) in the offshore species of *Avicennia marina* (Figure 3). These results concurred with the findings that *SLA* decreased while *WD* increased with salinity across mangroves in the Bangladesh Sundarbans [44]. We also noticed that *S. caseolaris* had significantly larger *SLA* than the introduced species of *S. apetala* (Figure 3a). This difference may come from the comparative advantage of the native species of *S. caseolaris* through long-term evolutionary adaptation to local hydro-environmental conditions to maximize leaf area and thus photosynthetic carbon assimilation [16].

**Figure A1:**
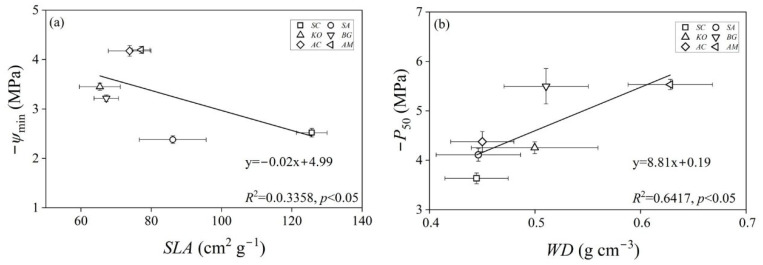
The linear regression relationships between the minimum leaf water potential (*ψ*_min_, −Mpa) and specific leaf area (*SLA*, cm^2^ g^−1^) (**a**) and the xylem water potential corresponding to 50% loss of hydraulic conductivity (*P*_50_, −Mpa) and wood density (*WD*, g cm^−3^) (**b**) across the six mangroves species. *SC*: *Sonneratia caseolaris*; *SA*: *Sonneratia apetala*; *KO*: *Kandelia obovata*; *BG*: *Bruguiera gymnorrhiza*; *AC*: *Aegiceras corniculatum*; *AM*: *Avicennia marina*.

In our present study, we observed that *SLA* and *WD* were marginally related to *ψ*_min_ (the minimum leaf water potential) and *P*_50_ (water potential corresponding to 50% loss in hydraulic conductivity) (Figure A1). However, *SLA* was not significantly different among other mangroves except for *S. caseolaris* and *S. apetala* (Figure 3a). Also, except for *A. marina*, the differences of *WD* were not significant among other mangrove species (Figure 3b). Such a dichotomy of *SLA* and *WD* of the studied mangroves further undermines their linear regression relationships with *ψ*_min_ and *P*_50_. These results corroborated the previous finding that *SLA* and *WD* were only weakly correlated with cross-species drought response patterns due to their loose connection to plant hydro-physiological processes [19]. Serra-Maluquer et al. [45] found that *SLA* and *WD* accounted for only 7% of drought-induced tree mortality variation across the globe. Powell et al. [46] showed that drought tolerance was independent of *WD* among mature Amazon rainforest trees. Therefore, despite the advantage of being easily measured for a large number of individual trees, *SLA* and *WD* per se do not necessarily presage plant vulnerability to drought or any other abiotic stress.

By contrast, hydraulic traits are generally considered to be more mechanistically linked to water use and transport in plants [47]. A large number of studies have demonstrated that hydraulic traits correlate tightly with plant vulnerability to drought [10,31]. We found that both *P*_50_ (water potential corresponding to 50% loss in hydraulic conductivity), *P*_88_ (water potential corresponding to 88% loss in hydraulic conductivity) and *ψ* (leaf water potential) decreased significantly along the salinity gradient from the onshore to offshore mangroves (Table 2 and Table A1). Jiang et al. [27] also observed that the *P*_50_ and *ψ*_min_ of the offshore salt-adapted mangroves were significantly lower than those of onshore species at Guangxi Beilun Estuary National Mangrove Reserve. It should also be noted that *Aegiceras corniculatum* had higher (less negative) *P*_x_ (including *P*_12_, *P*_50_ and *P*_88_) than *Bruguiera gymnorrhiza* in the present study (Table 2). This might be a result of the adaption of *A. corniculatum* to long-term inundation, being more available to water under the same or similar salinity. In natural conditions, *A. corniculatum* generally inhabits low tidal regions, while *B. gymnorrhiza* is commonly a mid–high tidal species [48,49].

The lower (more negative) *P*_50_ (<−5 Mpa) led to larger *HSM*s (hydraulic safety margins) (Figure 2b) for the offshore species of *A. marina* and *B. gymnorrhiza*, implying higher resistance to dehydration [11,46]. Yet, no obvious trends for *HSMs* were observed along the salinity gradient (Figure 2b). *A. corniculatum* and *K. obovate* had significantly narrower *HSM*s, signifying greater vulnerability to cavitation. Cavitated xylem conduits can be refilled at night through nocturnal sap flow, providing an effective compensation strategy for water deficits in these species [50]. On the other hand, although it is comparable in *ψ*_min_ (Table A1), *S. apetala* had a significantly larger *HSM* than *S. caseolaris* (Figure 2b). Photosynthesis and growth may be prioritized over drought resistance, restricting carbon investment to xylem and thus resulting in a less negative *P*_50_ and a narrower *HSM* for the native species of *S. caseolaris*.

*P*_x_ measures the water potential at which plants suffer a *x*% loss of hydraulic conductivity due to cavitation [32,51]; *HSM*s reflect the degree of conservatism of a plant’s hydraulic strategy [10,52]. The *S*_x_, which quantifies xylem embolism sensitivity to water potential changes, is also important. In alignment with the decline in *P*_50_ and *P*_88_, *S*_x_ (*S*_12,_ *S*_50_ and *S*_88_) also decreased along the salinity gradient from the onshore to offshore species (Table 2). Low *S*_x_ may serve as a buffer for plants to activate the *ABA* (abscisic acid) signaling pathway, inducing stomatal closure to reduce water loss through transpiration and preventing a further decline in *ψ* [53,54]. To sum up, it seems to be the case that there is a more gradual and slow progression of embolism than a sudden and rapid xylem embolization that dominates the acclimation and adaptation to salt in mangroves.

**Figure 4:**
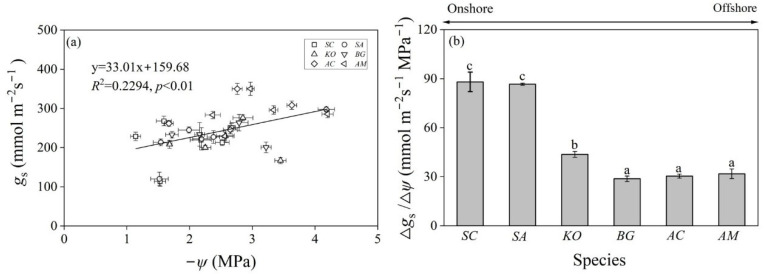
The linear regression relationship between leaf stomatal conductance (*g*_s_, mmol m^−2^ s^−1^) and water potential (*ψ*, −Mpa) (**a**) and the range ratio of stomatal conductance (△*g*_s_, mmol m^−2^ s^−1^) and water potential (△*ψ*, Mpa) (**b**) of the six mangrove species. Different letters indicate significant differences among species (mean ± SD, *n* = 3). *SC*: *Sonneratia caseolaris*; *SA*: *Sonneratia apetala*; *KO*: *Kandelia obovata*; *BG*: *Bruguiera gymnorrhiza*; *AC*: *Aegiceras corniculatum*; *AM*: *Avicennia marina*.

Hydraulic traits such as *P*_x_, *S*_x_, and *HSM*s mainly depict the capacity of the xylem of a plant species to resist cavitation and embolism [52]. Regulation of *g*_s_ (leaf stomatal conductance) represents another common strategy that plants adopt to cope with water deficiency under drought or saline conditions [55,56]. Species closing stomata rapidly can reduce transpiration water loss and maintain a relatively stable *ψ* in leaves (isohydry); by contrast, a loose control over *g*_s_ often leads to fluctuating *ψ* (anisohydry) [57]. The decreasing *ψ*_min_ (with a similar *ψ*_pre_) (Figure 2a and Table A1) indicated a gradual transition from isohydry to anisohydry for mangroves distributed along the onshore to offshore salinity gradient. Further analyses revealed that the onshore species of *S. caseolaris* and *S. Apetala* had significantly larger △*g_s_*/△*ψ* than the offshore species of *A. marina* and *A. corniculatum* (Figure 4b), supporting the argument that strict stomatal control over *ψ* prevailed in the inland mangrove species.

Across the six mangrove species studied here, *g*_s_ was significantly related to *ψ* (Figure 4a), suggesting that species with larger *g*_s_ exert less control over *ψ* (and thus leads to more negative *ψ*). Klein [22] compiled the response curves of *g*_s_ to *ψ* for 70 woody species from major forest biomes and found a continuum spectrum from anisohydry to isohydry. Other studies demonstrated that anisohydric species are more prone to be drought tolerant due to their embolism-resistant xylem and thick, dense leaves with lower turgor loss points [23,58]. In contrast, isohydric species often adopt a drought-avoidance strategy to prevent hydraulic failure during dry periods [59]. In the present study, we also observed smaller *SLA*, larger *WD*, and more negative *P*_50_ *and P*_88_ in the anisohydric species of *A. marina* and *A. corniculatum*. However, it is worth noting that a strict regulation of *ψ* is not necessarily associated with closed stomata and constrained carbon assimilation [60]. For a better prediction of plant responses to drought stress and a more reliable interpretation of community assemblage along salinity gradients, a prospective framework involves the integration of xylem hydraulics with stomatal behaviors as has been proposed by Klein [22] and Skelton et al. [61].

## 5. Conclusions

Based on the findings shown in the Section 3 and Section 4, and in correspondence to the hypotheses proposed in the introduction part, we make the following conclusions: (1) Hydraulic traits such as *P*_x_ (water potential corresponding to x% loss in hydraulic conductivity), *S*_x_ (slope of the hydraulic vulnerability curve), and *HSM*s (hydraulic safety margins) differentiated markedly among the studied mangrove species: species inhabiting high salinity sites tended to have more negative *P*_50_ (water potential corresponding to 50% loss in hydraulic conductivity) and *P*_88_ (water potential corresponding to 88% loss in hydraulic conductivity), and smaller *S*_x_, but *P*_12_ (water potential corresponding to 12% loss in hydraulic conductivity) and *HSM* displayed no salinity-associated trend. (2) Stomatal control over *ψ* (leaf water potential) decreased, and mangroves transitioned gradually from isohydry to anisohydry along the salinity gradient from the onshore to the offshore sites. (3) The onshore species of *S. caseolaris* and *S. apetala* had thinner leaves (larger *SLA* (specific leaf area )), while the offshore species of *A. marina* had significantly denser wood (larger *WD* (wood density)); both *SLA* and *WD* seemed to be more species-dependent rather than tolerance-related. More physiological features such as turgor loss points and a broader screening of mangrove species along topographically heterogenous coastal regions are needed to generalize our conclusions.

## Figures and Tables

**Table 1:** Symbols, descriptions, and units of measurement.

Symbols	Descriptions	Units
*K*	xylem hydraulic conductivity	kg m^−1^ s^−1^ Mpa^−1^
*K*_ini_	initial xylem hydraulic conductivity	kg m^−1^ s^−1^ Mpa^−1^
*K*_max_	maximum xylem hydraulic conductivity	kg m^−1^ s^−1^ Mpa^−1^
*P*_x_	xylem water potential corresponding to x% loss in hydraulic conductivity	Mpa
*P*_12_	xylem water potential corresponding to 12% loss in hydraulic conductivity	Mpa
*P*_50_	xylem water potential corresponding to 50% loss in hydraulic conductivity	Mpa
*P*_88_	xylem water potential corresponding to 88% loss in hydraulic conductivity	Mpa
*S*_x_	slope of the hydraulic vulnerability curve at *P*_x_	% Mpa^−1^
*S*_12_	slope of the hydraulic vulnerability curve at *P*_12_	% Mpa^−1^
*S*_50_	slope of the hydraulic vulnerability curve at *P*_50_	% Mpa^−1^
*S*_88_	slope of the hydraulic vulnerability curve at *P*_88_	% Mpa^−1^
*ψ*	leaf water potential	Mpa
*ψ*_min_	minimum leaf water potential	Mpa
△*ψ*	range from the minimum to the maximum leaf water potential	Mpa
*g*_s_	leaf stomatal conductance	mmol m^−2^ s^−1^
△*g*_s_	range from the minimum to the maximum leaf stomatal conductance	mmol m^−2^ s^−1^
*HSM*	hydraulic safety margin	Mpa
*SLA*	specific leaf area	cm^2^ g^−1^
*WD*	wood density	g cm^−3^

**Table 2:** Xylem water potential (Mpa) corresponding to hydraulic conductivity loss by 12% (*P*_12_), 50% (*P*_50_), and 88% (*P*_88_) and the slopes (% Mpa^−1^) of hydraulic vulnerability curves at *P*_12_ (*S*_12_), *P*_50_ (*S*_50_), and *P*_88_ (*S*_88_) for the six mangrove species. The 95% confidence intervals (CIs) for *P*_x_ and *S*_x_ were estimated by the bootstrap resampling approach with replacement (N = 1000). Different letters indicate significant differences among species. *SC*: *Sonneratia caseolaris*; *SA*: *Sonneratia apetala*; *KO*: *Kandelia obovata*; *BG*: *Bruguiera gymnorrhiza*; *AC*: *Aegiceras corniculatum*; *AM*: *Avicennia marina*.

Species	*P*_12_ (Mpa)	*P*_50_ (Mpa)	*P*_88_ (Mpa)	*S*_12_ (% Mpa^−1^)	*S*_50_ (% Mpa^−1^)	*S*_88_ (% Mpa^−1^)
*SC*	−1.59 [−1.40, −1.77] a	−3.64 [−3.46, −3.80] a	−6.31 [−5.93, −6.59] a	14.42 [13.90, 15.11] a	19.34 [17.97, 21.74] a	8.19 [7.31, 9.67] a
*SA*	−1.87 [−1.69, −1.9] ab	−4.10 [−3.94, −4.24] b	−6.90 [−6.58, −7.20] a	12.95 [12.56, 13.40] b	18.17 [16.89, 19.67] ab	7.93 [7.04, 8.93] a
*KO*	−1.66 [−1.51, −1.83] a	−4.22 [−4.06, −4.39] b	−7.81 [−7.35, −8.29] b	12.29 [11.85, 12.76] bc	14.91 [13.67, 16.33] c	5.91 [5.16, 6.85] b
*BG*	−2.65 [−2.27, −3.16] c	−5.47 [−5.09, −5.79] c	−8.83 [−8.33, −9.34] c	9.90 [9.47, 10.51] d	14.79 [13.09, 17.05] bc	6.73 [5.66, 8.38] ab
*AC*	−1.62 [−1.47, −1.76] a	−4.37 [−4.20, −4.51] b	−8.49 [−8.07, −8.90] bc	11.83 [11.45, 12.31] c	13.36 [12.41, 14.40] c	5.05 [4.50, 5.71] bc
*AM*	−2.07 [−1.86, −2.34] bc	−5.53 [−5.29, −5.79] c	−10.59 [−10.11, −11.19] d	9.34 [8.99, 9.77] d	10.79 [9.91, 11.61] d	4.14 [3.64, 4.77] c

**Table 3:** Diurnal leaf stomatal conductance (mmol m^−2^ s^−1^) of the six mangrove species. Different letters indicate significant differences among species (mean ± SD, *n* = 3). *SC*: *Sonneratia caseolaris*; *SA: Sonneratia apetala*; *KO: Kandelia obovate*; *BG: Bruguiera gymnorrhiza*; *AC*: *Aegiceras corniculatum*; *AM*: *Avicennia marina*.

Species/Time	06:00–08:00	8:00–10:00	10:00–13:00	13:00–15:00	15:00–17:00
*SC*	228.93 ± 10.75 bc	268.10 ± 12.21 ab	213.33 ± 7.63 bc	219.67 ± 5.85 a	113.93 ± 12.21 a
*SA*	213.57 ± 8.90 ab	245.13 ± 7.60 a	227.23 ± 16.07 c	222.70 ± 28.38 a	120.47 ± 17.00 a
*KO*	208.67 ± 10.35 a	276.27 ± 7.35 b	167.43 ± 8.14 a	230.27 ± 15.43 ab	200.40 ± 5.36 b
*BG*	233.13 ± 8.20 c	263.90 ± 21.11 ab	200.87 ± 13.66 b	251.73 ± 10.92 b	233.93 ± 30.05 c
*AC*	261.87 ± 6.89 d	350.23 ± 13.98 c	297.80 ± 6.15 d	308.30 ± 10.42 c	245.63 ± 10.23 c
*AM*	283.47 ± 9.30 e	350.37 ± 16.61 c	285.87 ± 9.49 d	296.13 ± 10.73 c	229.33 ± 10.45 c

## Data Availability

The original contributions presented in this study are included in the article. Further inquiries can be directed to the corresponding author.

## Appendix A

**Table A1:** Leaf water potential (*ψ*, Mpa) of the six mangrove species. Different letters indicate significant differences among species (mean ± SD, *n* = 3). *SC*: *Sonneratia caseolaris*; *SA*: *Sonneratia apetala*; *KO*: *Kandelia obovata*; *BG*: *Bruguiera gymnorrhiza*; *AC*: *Aegiceras corniculatum*; *AM*: *Avicennia marina*.

Species/Time	06:00–08:00	8:00–10:00	10:00–13:00	13:00–15:00	15:00–17:00	17:00–19:00
*SC*	−0.77 ± 0.02 b	−1.14 ± 0.08 d	−1.59 ± 0.12 c	−2.52 ± 0.11 d	−2.18 ± 0.17 d	−1.53 ± 0.09 c
*SA*	−0.94 ± 0.05 a	−1.54 ± 0.12 c	−1.99 ± 0.17 b	−2.38 ± 0.10 d	−2.19 ± 0.14 d	−1.52 ± 0.14 c
*KO*	−0.96 ± 0.06 a	−1.68 ± 0.04 bc	−2.85 ± 0.16 a	−3.45 ± 0.09 b	−2.57 ± 0.14 c	−2.25 ± 0.09 b
*BG*	−0.95 ± 0.03 a	−1.72 ± 0.1 b	−2.79 ± 0.14 a	−3.22 ± 0.08 c	−2.67 ± 0.06 c	−2.16 ± 0.08 b
*AC*	−0.74 ± 0.04 b	−1.67 ± 0.07 bc	−2.76 ± 0.08 a	−4.18 ± 0.13 a	−3.63 ± 0.08 a	−2.65 ± 0.11 a
*AM*	−0.40 ± 0.06 c	−2.37 ± 0.12 a	−2.97 ± 0.06 a	−4.20 ± 0.09 a	−3.34 ± 0.07 b	−2.56 ± 0.13 a

**Table A2:** Specific leaf area (*SLA*, cm^2^ g^−1^), wood density (*WD*, g cm^−3^), and hydraulic safety margin (*HSM*) of the six mangrove species. Different letters indicate significant differences among species (mean ± SD, *n* = 3–15). *SC*: *Sonneratia caseolaris*; *SA*: *Sonneratia apetala*; *KO*: *Kandelia obovata*; *BG*: *Bruguiera gymnorrhiza*; *AC*: *Aegiceras corniculatum*; *AM*: *Avicennia marina*.

Traits	SC	SA	KO	BG	AC	AM
*SLA*	125.68 ± 5.37 c	86.11 ± 11.65 b	65.36 ± 7.11 a	67.19 ± 4.27 a	73.84 ± 7.42 ab	77.12 ± 3.13 ab
*WD*	0.44 ± 0.04 a	0.45 ± 0.05 a	0.50 ± 0.06 a	0.51 ± 0.05 a	0.45 ± 0.09 a	0.63 ± 0.07 b
*HSM*	1.11 ± 0.03 c	1.73 ± 0.10 d	0.8 ± 0.06 b	2.28 ± 0.36 e	0.20 ± 0.10 a	1.34 ± 0.04 c
